# Gender Discrimination Among Academic Physicians

**DOI:** 10.1089/whr.2020.0031

**Published:** 2020-07-07

**Authors:** Candace J. Chow, Morgan M. Millar, Ana María López

**Affiliations:** Department of Internal Medicine, University of Utah School of Medicine, Salt Lake City, Utah, USA.

**Keywords:** discrimination, gender, identity

## Abstract

***Background:*** There is a growing body of literature showing that gender discrimination impacts physicians' work and life experiences. Impact on income, promotion, and parenthood has been documented. Based on these findings, we hypothesized that the experiences of academic physicians who identify as women or gender nonconforming would be different from their counterparts who are men. This survey study explores the influences of gender on academic physicians' experiences with discrimination in life and at work.

***Materials and Methods:*** In the spring of 2017, academic physicians (*n* = 752) at a medical school in the West were invited to participate in a survey that measured experiences with discrimination using the *Everyday Discrimination Scale* and additional items. We used a mixed-methods approach to analyze the data, employing chi square and *t*-tests to analyze quantitative data and modified content analysis to code open-ended responses.

***Results:*** The response rate was 24% (180/752). There was no significant difference between women and men in reported frequency of discrimination in everyday life (*p* = 0.474). However, women were significantly more likely than men to select gender as a reason for being treated differently in everyday life (*p* = 0.000) and report discrimination in the workplace (*p* < 0.000). Open-ended responses describing experiences of discrimination differed based on gender: women were twice as likely than men to report receiving negative treatment owing to gender. Finally, men discussed having gender privilege, whereas women discussed experiencing gender discrimination.

***Conclusions:*** This study contributes to the growing body of literature about how gender influences the experience of practicing medicine.

## Introduction

Gender inequality is widespread and has resulted in discrimination and violence against women throughout the world. The #MeToo movement, which started a decade ago but experienced a resurgence of popularity in 2017, has brought much needed attention to the issues of sexual harassment and gender-based discrimination.^1^ In addition to shedding light on its prevalence, it has prompted discussions about how to recognize, address, and prevent harassment and discrimination from occurring in public spaces, educational settings, and workplaces.

Workplace gender discrimination is not a new phenomenon. It is fostered by employees' bias,^2^ decisions to reinforce gendered norms even when they are counterproductive,^3,4^ and the interaction between gender stereotyping and institutional policies.^5^ Sexual harassment and gender discrimination in medicine are not new either, as research shows,^6,7^ and the #MeToo movement has given women physicians a new platform from which to share their experiences and call for change.^1^

Research provides numerous examples of discrimination against women physicians. They report being ignored, treated as outcasts, and treated differently from colleagues who are men.^7,8^ Women physicians earn less than peers who are men ^9–11^ and are not promoted to leadership roles at the same rates as physicians who are men.^10,12–14^ Because personal lives are hard to separate from work lives, it is not surprising that physicians who are parents also experience workplace discrimination. Although studies have focused on how women experience discrimination once they become mothers,^15^ citing a lack of formal maternity leave policies^16^ and insufficient postpartum accommodations,^17,18^ it is arguable that all physicians who are parents, regardless of gender, encounter discrimination when they engage in roles traditionally assigned to women, including child rearing. In sum, the literature documents the influence of gender identity on physicians' professional experience.

Given this research on women physicians' experiences, we sought to examine how academic physicians of all genders experience life and work. We hypothesized that one's life and work experiences would be influenced by one's gender, and that the experiences of women and gender nonconforming individuals would be different from that of men. To answer these questions, we surveyed academic physicians to (1) identify gender differences in reported instances of discrimination in life and at work and (2) understand how participants of different genders classify and describe experiences of discrimination.

## Materials and Methods

### Study population

A sample of academic physicians at our medical school, located in the Mountain West region of the United States, were invited to take part in a survey about their identities and experiences in medicine. This sample included all the tenure track faculty with medical (MD/DO) degrees (*n* = 376) and an equal number (*n* = 376) of clinical career track faculty. The latter were randomly selected and asked to participate. A total of 752 physicians were invited to participate.

### Survey design and administration

The survey was administered using REDCap, a secure, web-based application^19^ in February–April 2017. We sent four email messages to request participation. Responses remained anonymous. A consent cover letter was used to consent participants. The university's Institutional Review Board deemed this study exempt.

**FIG. 1.**
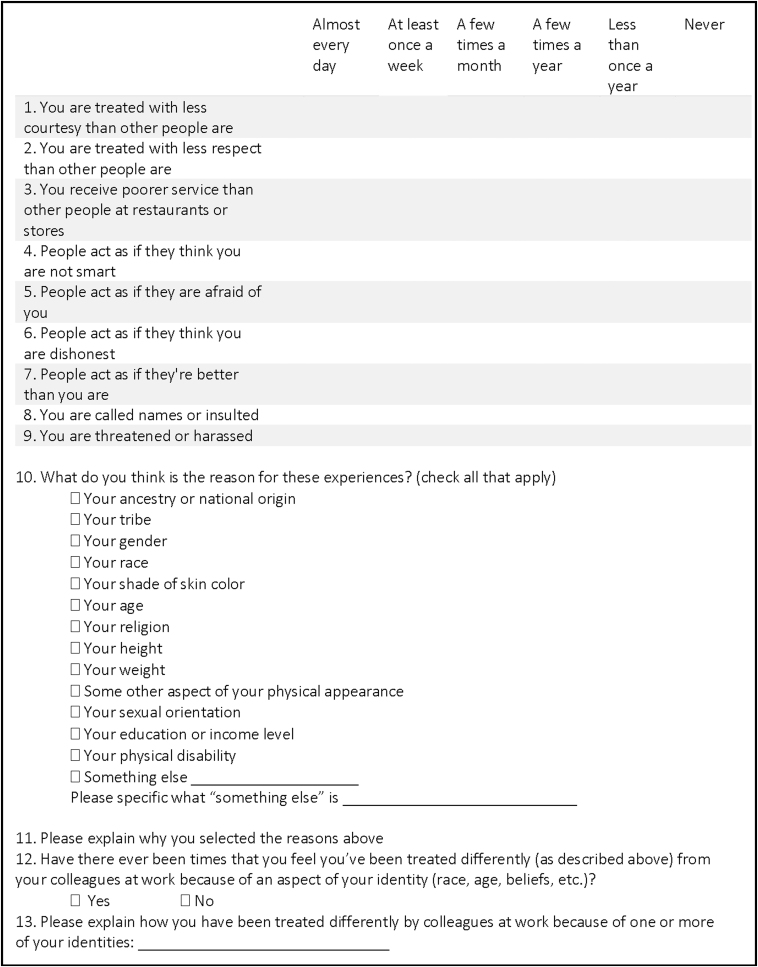
Life experiences section of survey.

The survey was created using a combination of existing instruments and newly developed items (Fig. 1). The survey also included demographic questions. Reported instances of discrimination in life (research question 1) were captured using the *Everyday Discrimination Scale* that measures “chronic, routine, and relatively minor experiences of unfair treatment.”^20^ This previously developed, psychometrically tested scale includes nine items to measure how often participants experience discrimination, broadly defined, in daily life (*e.g.*, “You are treated with less courtesy than other people are”). Responses are collected using a six-point Likert scale, ranging from almost everyday to never and summary scores are calculated. We developed an additional question (item 12) to ascertain instances of discrimination *at work specifically*. It asked whether or not participants have been treated differently from colleagues at work because of some aspect of their identity (research question 1).

Classification and description of experiences with discrimination in life (research question 2) were captured using the 10th, follow-up question on the *Everyday Discrimination Scale* that asks participants to select the perceived reasons for experiencing (or not experiencing, in the case that “never” is selected for all scale items) discrimination, which includes gender, race, age, and 11 other identities or attributes. We added additional open-ended items that asked participants to “Please explain why you selected the reasons above” (item 11) and “Please explain how you have been treated differently by colleagues at work because of one or more of your identities” (item 13) to understand how participants classified and described experiences with discrimination at work.

### Methods of analysis

Because survey responses yielded both closed and open-ended data, quantitative and qualitative methods of analysis were used, using Stata MP 13.1 (Stata Corp., College Station, TX) and Dedoose Version 7.0.23 (SocioCultural Research Consultants, LLC, Los Angeles, CA), respectively. Descriptive statistics were used to summarize responses to demographic questions. *Everyday Discrimination Scale* scores were calculated by summing responses to the nine Likert items to produce a composite score. Instances of discrimination at work (item 12) were coded as yes/no and are reported descriptively. Gender differences in responses were compared using chi square tests and *t*-tests as appropriate. Responses to the item (10) asking for the reason participants experience discrimination in everday life were dichotomized (1 = gender, 0 = other) and tallied. Responses to open-ended questions regarding reasons for being treated differently (items 11 and 13) were analyzed using modified content analysis^21^ and coded for themes by the first and second authors.

## Results

### Demographics

A total of 180 physicians completed the survey (response rate of 23.9%). This rate is consistent with other surveys of physicians.^8,15^ Descriptive statistics are given in Table 1. Eighty participants (44.4%) identified as women and none identified as gender nonconforming. Twenty-four participants (13.3%) identified as a person of a minoritized racial or ethnic group. Because the sample of racially/ethnically minoritized physicians was small, we did not account for race or ethnicity in analysis. Of all specialties, pediatrics was most common (*n* = 55, 30.7%), and the largest group of participants (*n* = 61, 34.1%) were those who had completed postgraduate studies for more than 20 years.

**Table 1.** Demographic Characteristics of Participants in a Survey of Academic Physicians

Characteristics	All participants		Men		Women	
	N	%	n	%	n	%
Total	180	100				
Gender						
Woman	80	44.4				
Man	100	55.6				
Race/ethnicity						
Minoritized race/ethnicity	24	13.3	17	17.0	7	8.8
Non-Hispanic white	156	86.7	83	83.0	73	91.3
Year of high school graduation						
1969 or earlier	15	8.8	12	12.6	3	4.0
1970–1979	39	22.8	22	23.2	17	22.4
1980–1989	42	24.6	27	28.4	15	19.7
1990–1999	57	33.3	26	27.4	31	40.8
2000 or later	18	10.5	8	8.4	10	13.2
Department						
Pediatrics	55	30.7	38	38.4	30	37.5
Internal medicine	40	22.4	22	22.2	33	41.3
Surgery	16	8.9	27	27.3	13	16.3
Other	68	38.0	12	12.1	4	5.0
Years as attending physician						
5 or fewer	51	28.5	25	25.3	26	32.5
6–10	31	17.3	15	15.2	16	20.0
11–15	20	11.2	9	9.1	11	13.8
16–20	16	8.9	11	11.1	5	6.3
More than 20	61	34.1	39	39.4	22	27.5

Percentages based on total number of respondents providing a response to each individual question.

### Gender differences in reported instances of discrimination

We assessed whether gender is associated with instances of reported discrimination in life using the *Everyday Discrimination Scale* (items 9 and 10) and in work using an additional item (question 12) asking for experiences of discrimination at work (Table 2). There was no significant difference between women and men in reported frequency of discrimination in everyday life based on average *Everyday Discrimination Scale* scores (*p* = 0.474). However, in response to the question asking for the perceived reasons for everyday discrimination experiences (item 10), of those who reported at least one reason for experiences of everyday discrimination, 87 (53.4%) indicated gender. Women were significantly more likely than men to select gender as a reason for being treated differently in everyday life; 84.0% of women noted gender as a reason, whereas only 27.3% of men did so (*p* = 0.000). When asked about experiencing discrimination at work specifically (item 12), women were significantly more likely than men to report experiencing discrimination in the workplace (62.0% women vs. 34.0% of men, *p* < 0.000).

**Table 2.** Gender Differences in Experiences of Discrimination in Everyday Life and at Work

Measure of discrimination	All participants	Men	Women	p
Everyday discrimination scale, mean (standard deviation)	18.8 (5.9)	18.5 (6.3)	19.1 (5.5)	0.474
Reason for discrimination = gender				
Yes, *n* (%)	76 (46.6)	24 (27.3)	63 (84.0)	**0.000**
No, *n* (%)	87 (53.4)	64 (72.7)	12 (16.0)	
Experienced discrimination at work				
Yes, *n* (%)	83 (46.4)	34 (34.0)	49 (62.0)	**0.000**
No, *n* (%)	96 (53.6)	66 (66.0)	30 (38.0)	

Bold denotes statistical significance *p* < 0.05.

Percentages based on total number of participants providing a response to each individual question.

### Classification of experiences of discrimination

We used responses to items 11 and 13 to understand how participants classified experiences of discrimination. Gender was the most common reason given in both instances. Women were more than three times as likely than men to list gender as the reason for experiences of discrimination in everyday life, and six times as likely as men to mention gender as the reason for their experiences of discrimination at work.

A total of 105 (58%) participants provided responses (Table 3) to the question (item 11) that asked participants to explain why they selected the reason(s) for how they are treated in their everyday life. Responses describing experiences of discrimination differed based on gender. Although 17 (16.2%) of the 105 participants reported that their gender identity resulted in positive treatment (*e.g.*, it was a benefit), 63 (60.0%) reported that their identities resulted in negative treatment (*e.g.*, it resulted in discrimination). The remaining 25 (23.8%) reported responses that were neither positive nor negative. Fifteen of the 17 participants who reported positive treatment were men. Conversely, women were more likely than men to report negative treatment: 87.8% of women's responses indicated negative treatment, whereas only 35.7% of men's responses reported negative treatment (*p* = 0.000).

**Table 3.** Classification of Open-Ended Responses Describing How Participants Are Treated Differently in Everyday Life and at Work, by Gender, from a Survey of Academic Physicians

	All participants	Women	Men	p
	n (%)	n (%)	n (%)	
Treated differently in everyday life				
Positive reaction (*e.g.*, treated better)	17 (16.2)	2 (4.1)	15 (26.8)	**0.000**
Negative reaction (*e.g.*, treated worse)	63 (60.0)	43 (87.8)	20 (35.7)	
Neutral reaction	25 (23.8)	4 (8.2)	21 (37.5)	
Treated differently at work				
Positive reaction (*e.g.*, treated better)	7 (9.7)	1 (2.3)	6 (21.4)	**0.027**
Negative reaction (*e.g.*, treated worse)	55 (76.4)	36 (81.8)	19 (67.9)	
Neutral reaction	10 (13.9)	7 (15.9)	3 (10.7)	
Reasons for differential treatment				
Gender (in everyday life)	47 (44.8)	37 (75.5)	10 (17.9)	**0.000**
Other (in everyday life)	58 (55.2)	12 (24.5)	46 (82.1)	
Gender (at work)	42 (58.3)	36 (81.8)	6 (21.4)	**0.000**
Other (at work)	30 (41.7)	8 (18.2)	22 (78.6)	

Bold denotes statistical significance *p* < 0.05.

The second open-ended question (item 13) asked participants to explain how they have been treated differently by colleagues at work because of one or more of their identities. For this item, 40% of participants provided responses (*n* = 72). Whereas 7 (9.7%) reported that they received positive treatment based on their identities, 55 (76.4%) reported receiving negative treatment. Of the participants who reported positive treatment, 6 (21.4%) were men. Among all responses provided by women, 36 (81.8%) reported negative treatment, whereas only 19 (67.9%) of men's responses indicated negative treatment (*p* = 0.027).

We also used open-ended responses to identify participants' perceptions of what aspects of their identities were related to their experiences of discrimination. Gender was the most common reason given in both instances. Women were more than three times as likely than men to list gender as the reason for experiences of discrimination in everyday life (*p* = 0.000), and six times as likely as men to mention gender as the reason for their experiences of discrimination at work (*p* = 0.000).

### Thematic descriptions of experiences with gender

Responses to these questions (items 11 and 13) were also thematically coded to understand how participants experienced gender in everyday life and at work (Table 4). Among women, some mentioned experiencing gender discrimination in their personal lives in response to the first question: “…going to the car mechanic or car shopping, I feel I am not treated with the same respect as a man would receive.” However, most (65%) women participants talked about how they were treated differently at work for both questions, although only the second question specifically asked about the workplace.

**Table 4.** Themes from Open-Ended Comments

Gender identity	Reaction	Reason	Exemplar quotes
Woman	Positive	Gender	Very rarely, I have been treated with less respect as a woman
Woman	Positive	Nongender related	I feel that I have advantages because I am well educated, have frequently been deferred to because of my height even if that situation would not call for that (being the medical student with an attending who is smaller than me and the patient asks me a question). I know that society gives white people advantages that I probably am not fully aware of. But also my education and thus communication skills allow me other advantages
			As a physician, I am generally treated with more respect/deference than nonmedical professionals I work with closely. Within a medical hierarchy, however, there are occasions that my role is perceived to be less important (“social medicine”)
	Negative	Gender (men are generally treated better)	I feel there is systematic gender discrimination in [my state], and my workplace is no different
		Gender (women receive less credit)	Opinions discounted because I'm female
		Gender (women hold less authority)	Even though I am in position of authority I feel like people often bypass me and go to the men in the division for support, advice, *etc*
		Gender (women have fewer opportunities for advancement and compensation)	Women have fewer advancement opportunities, make somewhat less money
		Gender (women have child-rearing responsibilities)	Opportunities for certain types of work are not available due to me prioritizing my family when I'm at home
	Negative	Nongender related	Most likely to experience lack of courtesy and disrespect from patients/families in the work setting
			[my state's] culture does not feel very inclusive. If you move here as an outsider, it is still hard to feel a part of the community
			Because I am a junior faculty, I am not free as much to express my opinions or my opinions are not weighted as seriously
	Neither positive nor negative	Gender	As a female pulmonary/critical care physician, I experience some assumptions that seem to be made because of my gender. Patients often assume that I am a nurse, which I actually do not find to be offensive. Other health care professionals/staff treat female physicians differently from male physicians. It is debatable whether this is a bad or good thing, however; often this seems to result in a lower power distance index and greater communication, as other staff seem less intimidated, and more willing to question medical decisions/orders because I am female
		Nongender related	These events happen so infrequently, it is difficult to explain why
Man	Positive	Gender	…I don't think I experience this much as I am a white male
			I clearly have privilege as a white male doctor over the women and minorities [I] work with
	Positive	Nongender related	I essentially never feel that I am discriminated against in [my state]
			With advancing age I seem to get more respect from others
			Just a best guess but other than a few extremely wealthy people, this is not an issue for me and I know I am fortunate in this regard
	Negative	Gender	Sometimes women seem to be hesitant/cautious of a male in an isolated setting (*e.g.*, on a trail). this is totally understandable
	Negative	Nongender related	Psychiatrists are not well-respected physicians
			I think overweight people are looked at as being that way because they are lazy, lack self-control, or a combination of other things
			Sometimes I think I am treated differently in this community because I am not [part of the dominant religion]
			People will infrequently be rude or hurtful, but I usually view that as a being based on the other person behaving improperly, and not being a direct result of my own identity or physical attributes
			Sometimes my faith has been the subject of negative comments by others
	Neither positive nor negative	Gender	I am a tall, white, educated, middle-aged, male physician in some positions of authority with progressive/liberal viewpoints
		Nongender related	My age, dress, and youthful looking appearance
			Having a large family

The men in our study talked about experiencing negative treatment for a variety of reasons, including religion, level of attainment, and overall appearance. However, when it came to treatment specifically related to gender, all but two men (one of whom happened to self-identify as White or biracial [Asian and White]) reported experiencing privilege. These men who reported experiencing discrimination attributed it to having an imposing physical appearance: “I am a large man with a beard and sometimes people are fearful of me when they see [me].” On the contrary, women reported receiving differential treatment because of gender in five ways: men are generally treated better, women receive less credit, women hold less authority, women have fewer opportunities for advancement and compensation, and women being treated differently because they have family and child-rearing responsibilities. Because the themes were the same for both everyday life and work, we report on them together.

#### Men have privilege

Men acknowledged their gender privilege, explaining: “I receive preferential treatment,” “I think I'm in the group, that is, least likely to suffer,” and “Relative expectations of my responsibilities to my family and my work have been different for me as a male (husband and father) than for my spouse (female, wife, and mother) or the female members in my division.”

#### Men are generally treated better

Some women talked about treatment in a global, overarching way: “It is still a male dominated world.” Others talked about experiencing bias in a general way: “I feel there is still a good deal of gender bias in medicine,” and “I feel there is systematic gender discrimination in [this state], and my workplace is no different.”

#### Women receive less credit

Women spoke about receiving less recognition for ideas. One noted, “I've been in many physician groups where the comments of women are ignored and the same comments by men are celebrated.” Others explained that women receive less credit for their accomplishments: “talents and skills [are] not recognized despite credentials” and “I had to prove myself to the other specialists, surgeons, *etc.*”

#### Women hold less authority

Women explained that they have less authority in the workplace. Sometimes it is from patients: “Several times I have had patients think that either male medical students or older students/residents were my attending physician when roles were reversed.” Other times this came from colleagues: “Sometimes patients, staff, and colleagues are less likely to accept my influence or opinion because of my gender.”

#### Women have fewer opportunities for advancement and compensation

Women perceived fewer opportunities for promotion: “Women do not seem to be offered the same opportunities for advancement.” Participants also noted that gender parity in pay was lacking: “…we had a recent departmental review…women in our department are paid less than the men.” Another participant noted, “Women are frequently assigned additional responsibilities without additional time or compensation, while the time of men seems to be more frequently compensated.”

#### Women have family and child-rearing responsibilities

Finally, women recalled being treated differently because of responsibilities at home: “…as a female who is a mom and believes, that is, the most important calling in life I am treated differently in my job.” Another participant noted that their colleagues had said things to them like, “you need to get home early to your kids.”

## Discussion

This study sought to (1) identify gender differences in reported instances of discrimination in life and at work and (2) understand how participants of different genders classify and describe experiences of discrimination. There were no statistically significant differences between men and women's reported frequency of discrimination in everyday life. Although women were not significantly more likely than men to report discrimination in daily life, women were three times as likely as men to see their gender as a reason for everyday experiences of discrimination. In the workplace, women were twice as likely as men to attribute discrimination to gender. This is consistent with previous research^7,9,15,17,22^ that has found that women physicians experience gender discrimination in the workplace. This is especially troubling given that gender discrimination predicts burnout among women physicians,^15^ which in turn can lead to women physicians leaving the workforce. Efforts to diversify the workforce and increase women in positions of leadership will fall flat if women are hired into an environment where they will be subject to discrimination—an environment that neither supports their survival much less their success.

Women were twice as likely as men to report negative reasons for being treated differently in everyday life, and nearly 1.5 times to report this at work. Our qualitative analysis revealed that this is because men saw their gender as a reason for experiencing positive treatment, whereas women saw their gender as the reason for experienced negative treatment. Aside from one exception in which a man reported being discriminated owing to physical appearance, men described instances in which their gender gave them privilege. Some women in our study reported that men are treated better in a global sense in that society and the workplace generally favor men over women. Previous research has found that women describe the academy as being a system that values men over women^22^ and one in which women are more likely than men to report being bullied.^23^ In addition to feeling gender discrimination in a global sense, women in our study reported receiving less credit for their work. Rouse et al.^23^ also found that women in their study reported a lack of recognition for the work they did. Also similar to previous findings, women in our study reported holding less authority,^23,24^ having fewer opportunities for advancement,^13,14,18,24,25^ and being treated differently because of childrearing responsibilities.^18,24^

Overall, our findings indicate that although women physicians do not report more frequent everyday discrimination compared with men, when they are asked about ways in which they experience discrimination, they attribute it to gender. One possible explanation for this finding is the racial composition of the women in our study. Although some of the women identified as racially/ethnically minoritized (9%), >91% identified as White. Race may serve as a protective factor for these women—protecting them against racial/ethnic discrimination, and rendering them subject mainly to gender discrimination, which for one reason or another, is not occurring with high frequency. Another reason for our findings may be that participants were not asked to provide reasons for experiences of discrimination until the end, so participants may not have been primed to think about experiences of gender discrimination while answering the closed-ended questions section of the *Everyday Discrimination Scale*, but they were primed to think about gender when they answered questions about work experiences. A final explanation is that women experience gender discrimination because medicine is an historically male-dominated field.^26,27^ Although the gender composition of physicians is changing, the change is slow, especially at the leadership level.^22,25,28^ Moreover, because it is becoming more acceptable to openly discuss issues of gender discrimination and sexual harassment in the workplace, it is possible that the participants in our study attributed discrimination owing to gender because it is the most socially acceptable answer.

As other scholars who have conducted research on gender discrimination in academic medicine have suggested, changes are needed to make a difference for women physicians.^28,29^ Programs specifically geared to equip women to succeed^29^ are certainly necessary, and mentoring women to think about leadership and success the way that men do^25,28^ might be one plausible approach. However, addressing campus climate change in addition to supporting women is necessary because addressing gender discrimination requires a systemic institutional approach.^29^ It has been suggested that initiatives that encourage men to shoulder equal responsibility for child rearing could ease the burden placed on women and also make men more understanding of the experience of their counterparts.^8^ Another approach is to develop campus-wide programs that focus on developing men faculty as allies and advocates so that they can help to bring about institutional change.^30,31^

This study has its limitations. Because the majority of respondents identified as White, we were unable to assess whether race and ethnicity or the interaction between gender and race/ethnicity could also be factors for experiences of discrimination. Likewise, although the majority of men participants, who also happened to be White or biracial, reported experiencing gender privilege, we were not able to assess whether men from different racial backgrounds are subject to gender or gender and racial discrimination. Future directions could include investigating whether other demographic factors, such as religion and socioeconomic status, or the interaction between such factors and gender, predict experiences of discrimination in everyday life and work.

## Conclusions

This research contributes to the growing evidence that women and men physicians employed at an academic health center experience medical careers quite differently.^32^ There are no differences in the frequency with which women and men physicians report experiencing discrimination in everyday life. However, findings show that women physicians in our study report experiencing discrimination at work more frequently than physicians who are men, but that discrimination is not a part of their everyday experience. However, when asked why discrimination occurs, women are more likely than men to report experiencing discrimination because of their gender. Future projects are warranted to investigate what interventions are successful at addressing women's experiences with inequality in medicine.
